# Inhibitor of CBP Histone Acetyltransferase Downregulates p53 Activation and Facilitates Methylation at Lysine 27 on Histone H3

**DOI:** 10.3390/molecules23081930

**Published:** 2018-08-02

**Authors:** Adam S. Vincek, Jigneshkumar Patel, Anbalagan Jaganathan, Antonia Green, Valerie Pierre-Louis, Vimal Arora, Jill Rehmann, Mihaly Mezei, Ming-Ming Zhou, Michael Ohlmeyer, Shiraz Mujtaba

**Affiliations:** 1 Department of Pharmacological Sciences, Icahn School of Medicine at Mount Sinai, New York, NY 10029, USA; adam.vincek@mssm.edu (A.S.V.); jigneshkumar.patel@bcm.edu (J.P.); ajaganat@cshl.edu (A.J.); mihaly.mezei@mssm.edu (M.M.); ming-ming.zhou@mssm.edu (M.-M.Z.); michael.ohlmeyer@mssm.edu (M.O.); 2 Department of Genetics and Genomic Sciences, Icahn School of Medicine at Mount Sinai, New York, NY 10029, USA; 3 One Bungtown Rd, Cold Spring Harbor Laboratories, Cold Spring Harbor, NY 11724, USA; 4 Department of Physical Science, St. Joseph’s College, 245 Clinton Avenue, Brooklyn, NY 11205, USA; antoniagreen13@gmail.com (A.G.); vpierrelouis90@gmail.com (V.P.-L.); jrehmann@sjcny.edu (J.R.); 5 Department of Biology, City University of New York, Medgar Evers College, Brooklyn, NY 11225, USA; Vimal.Arora@student.mec.cuny.edu

**Keywords:** lysine acetylation, transcriptional coactivator CBP, histone acetyltransferase, *cy*-*C*_5_-curcuminoids, p53 acetylation, histone acetylation, methylation

## Abstract

Tumor suppressor p53-directed apoptosis triggers loss of normal cells, which contributes to the side-effects from anticancer therapies. Thus, small molecules with potential to downregulate the activation of p53 could minimize pathology emerging from anticancer therapies. Acetylation of p53 by the histone acetyltransferase (HAT) domain is the hallmark of coactivator CREB-binding protein (CBP) epigenetic function. During genotoxic stress, CBP HAT-mediated acetylation is essential for the activation of p53 to transcriptionally govern target genes, which control cellular responses. Here, we present a small molecule, NiCur, which blocks CBP HAT activity and downregulates p53 activation upon genotoxic stress. Computational modeling reveals that NiCur docks into the active site of CBP HAT. On *CDKN1A* promoter, the recruitment of p53 as well as RNA Polymerase II and levels of acetylation on histone H3 were diminished by NiCur. Specifically, NiCur reduces the levels of acetylation at lysine 27 on histone H3, which concomitantly increases the levels of *tri*methylation at lysine 27. Finally, NiCur attenuates p53-directed apoptosis by inhibiting the Caspase 3 activity and cleavage of Poly (ADP-ribose) polymerase (PARP) in normal gastrointestinal epithelial cells. Collectively, NiCur demonstrates the potential to reprogram the chromatin landscape and modulate biological outcomes of CBP-mediated acetylation under normal and disease conditions.

## 1. Introduction

Targeting tumor cells by anticancer therapies, which are effective in controlling the growth of tumors, also generate severe adverse effects [1]. Massive cellular loss is one of the major side-effects of anticancer therapies, which eventually damage the architecture of normal tissues, including gastrointestinal epithelial lining, bone marrow, and hair follicles [2,3]. Ultimately, radiation-induced pathology within normal tissues deteriorates the physical health of cancer patients [2,3]. Thus, current therapeutic approaches need to expand for safeguarding the normal tissues from the pathology emerging due to aggressive anticancer treatments [4]. First generation small molecules, such as pifthrin (PFT)-α, inhibited p53 function in cellular and mice models [5]. However, the structural basis for selectivity and the molecular targets of PFT-α remain to be completely understood [6]. Similarly, the mechanistic underpinnings of amifostine activity, which is being used for preventing cellular loss and tissue damage from radiation, are less understood [2,3]. During many stress conditions, including DNA damage, viral infections, and inflammatory disorders, lysine acetylation of chromatin-associated proteins is one of the major mechanisms that regulate transcriptional activities [7,8,9]. Subsequently, acetylation-mediated transcriptional regulation modulates cell-fate decisions and cellular responses [10]. Given the central role of lysine acetylation in transcriptional regulation and cellular processes, coactivators mediating acetylation of lysine residues have been a target for developing new small molecules to thwart diseases, like HIV/AIDS and cancers [11]. Further, molecular pathways emerging due to site-specific lysine acetylation have been the focus of recent drug discovery program for many disease models due to promising treatment outcomes [12]. The present study is based on the notion that blocking lysine acetylation could prevent radiation-induced pathology in normal tissues, which would ameliorate the side effects of anticancer treatments.

During a multitude of stress conditions, transcriptional activation of p53 controls cellular proliferation by inducing DNA repair, cell cycle arrest, or apoptosis [13]. Stress-induced site-specific post-translational modifications, such as phosphorylation, acetylation, and ubiquitination, are essential for regulating the transcriptional activities of p53 [14]. Phosphorylation of p53 at serine 15 (p53S15p) relieves p53 from the negative regulation by mouse double minute 2 homolog [15]. Then, acetylation of p53 at lysine 382 (p53K382ac) by transcriptional coactivator CREB-Binding Protein (CBP) serves as the docking site for the acetyl-lysine-binding module, bromodomain (BRD) of CBP. Subsequently, p53K382ac/CBP BRD interaction activates the transcription of *Cyclin Dependent Kinase Inhibitor 1A* or *p21* (*CDKN1A/p21*), which induces growth arrest in DNA damaged cells [16,17,18]. In addition to the p53K382ac/CBP BRD interaction, kinase-inducible and Transcription Adaptor putative Zinc finger domains of CBP interact with the transactivation domain of p53 [19,20]. Although a small molecule activating p53 offers an attractive path for cancer treatment, pharmacological blocking of p53 provides a compelling rationale for developing the inhibitors of p53 in ischemia as well as in neurological disorders and in downregulating mutant *p53,* which could be helpful in cancers [16,21]. Moreover, downregulation of p53 functions using small molecules, including PFT-α and amifostine, have been reported to reduce radiation-induced pathology, particularly, in epithelial linings of gastrointestinal tissues [21]. Together, blocking CBP HAT activity offers a valid target for rationale-based designing of chemical modulators, which could regulate p53 transcriptional functions.

The ubiquitous transcriptional coactivator CBP is involved in the regulation of growth, development, and differentiation, as well as during the modulation of cellular response to DNA damage, viral infections, and inflammation [7,8,22]. CBP regulates downstream gene activity by imprinting site-specific acetylation marks on the lysine residues of the histone proteins within chromatin and chromatin-associated proteins in response to changes in extracellular environment [10]. Besides post-translational modification of p53, acetylation of lysine 27 on histone H3 (H3K27ac) by CBP HAT serves as a marker for gene activation [23]. However, *tri*methylation of the H3K27 site (H3K27me3) by Enhancer of zeste homolog 2 (EZH2), a histone methyltransferase, which is one of the components of the *Polycomb* repressor complex (PRC), leads to gene silencing [24]. Thus, inhibition of CBP HAT has the potential to modulate the levels of H3K27ac versus H3K27me3 on the chromatin of a gene promoter causing chromatin remodeling leading to gene activation versus gene silencing. Further, a study using human melanocytes showed that downregulation of CBP HAT activity inhibited growth and induced cellular senescence [25]. Besides, depending upon the cellular context, CBP HAT activity is required for the G1/S transition of the cell cycle [26]. Furthermore, genes for the monocytic leukemia zinc finger protein and p300/CBP HAT domain undergo fusion by chromosomal translocation causing myeloid leukemia [27]. Together, CBP HAT could serve as a valuable pharmacological target to develop small molecules for minimizing anticancer therapy-induced pathology in normal tissues as well as intercepting oncogenic fusion proteins in diseases, such as leukemia.

Curcumin is the main active ingredient of turmeric, which is a powdered *Curcuma longa* L*. rhizome* that is used as a traditional spice, pigment, and medicine in Asia [28]. A mono-carbonyl analog of curcumin is *C*_5_-curcumin, a naturally occurring component in *Curcuma domestica V. rhizomes* [29]. *C*_5_-Curcumin analogs have been widely reported to possess improved pharmacokinetic activity profiles, as well as structural stability, while maintaining low toxicity relative to curcumin [29,30]. Structurally modified *C*_5_-curcumin analogs, acyclic and cyclic, such as (3*E*,5*E*)-3,5-bis[(2-fluorophenyl)methylene]-4-piperidinone (EF24) and a nitrile-curcuminoid were previously demonstrated to exhibit anti-inflammatory [30], anti-angiogenic, and anticancer activities [31]. However, the underlying molecular mechanisms, which direct the cellular effects of curcumin, are still not fully clear. Curcumin activates the expression of NF-E2-related factor 2 required for free radical scavenging, detoxification of xenobiotics, and maintenance of redox potential [32]. *C*_5_-curcumin analogs have also been tested for inhibition of histone lysine methyltransferases [33] and of sirtuins [34]. Thus, curcumin could serve as a chemical scaffold to design diverse chemical modulators of proteins that engage in epigenetically-regulated cellular functions. Intercepting acetylation-directed mechanisms could prove valuable in pathological settings because an acetylated-lysine moiety could recruit either an activator or a repressor complex, which could potentially exacerbate disease pathogenesis. Most importantly, these small molecule chemical agents could serve as a valuable research tool to understand the complex underlying mechanisms of transcription regulation in normal and disease situations. In this study, a two-pronged strategy was undertaken to synthesize analogs of *cy*-*C*_5_-curcuminoids. Then, the most potent CBP HAT inhibitor was tested for modulating gene regulatory functions of CBP.

## 2. Results

### 2.1. Structure–Activity Relationship Analysis of cy-C_5_-Curcuminoid Analogs

**Figure 1:**
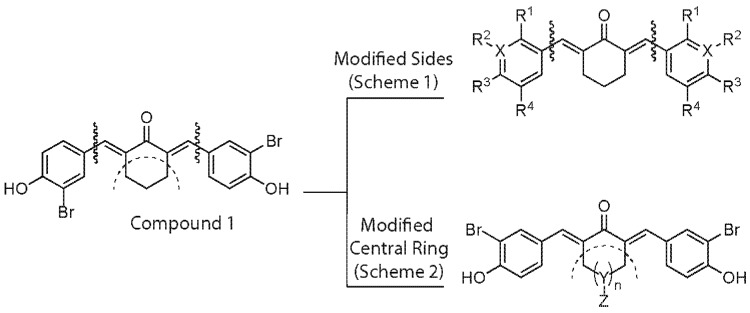
Two-pronged approach to synthesize chemical analogues of a cy-*C*_5_-curcuminoid compound **1**. In Scheme 1, Montmorillonite K-10 clay was utilized with microwave assisted organic synthesis (MAOS) to synthetically generate the analogs of *cy-C*_5_-curcuminoid with modification on sides (K-10/MAOS). In Scheme 2, synthetic methods were used to modify the central ring of *cy*-*C*_5_-curcuminoid for generating new analogs.

A two-pronged strategy was devised to develop a potent CBP HAT inhibitor by synthesizing analogs of *cy*-*C*_5_-curcuminoid (Figure 1).

**Scheme 1:**
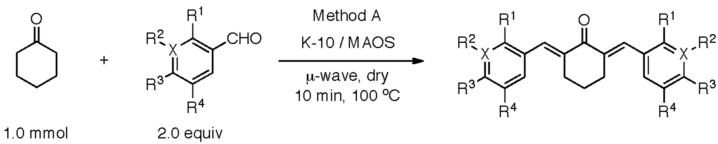
K-10/MAOS were used to synthetically generate the new analogs of *cy-C*_5_-curcumin with modified sides.

Scheme 1 depicts the synthesis of new analogs, which were mainly altered on the side structures of compound **1** (Figure 1). Table 1 lists the inhibitory effect of each compound, which was determined by a luciferase-based reporter assay that involves Doxorubicin (Dox)-mediated activation of p53 response element. Supplementary Table S1 shows the raw luciferase data for calculating the percentage inhibition of each compound. While curcumin attenuated p53 activation by 61%, the previously described *cy*-*C*_5_-curcumin analog **1** was approximately 50% less active than the curcumin. Notably, the m-CN compound **2** inhibited the activation of p53 most significantly. The methoxy analog **3**-mediated inhibition of p53 activation was similar to that of curcumin. Comparatively, p-OH **7** and di-m-Me-p-OH **6** analogs were more effective toward attenuating p53 activation than di-m-Me **5** and o-Me **4** analogs, which indicated that their activity was independent of phenolic hydroxy functionality. The activity of four halogenated analogs, including the o-F **8**, o-Br **9**, m-Br **10**, and p-Br **11** ranged from very low to high inhibitory potential. The electron withdrawing m-bromo analog **10** was highly potent when compared to the other halogenated analogs, such as the previously described analog and curcumin. Analogs meta, and free phenolic hydroxy functionality **2**, **3**, **10**, and **12** displayed increased potency for inhibiting the p53 activation. While both m-CN **2** and m-pyridinyl **12** demonstrated the highest degree of inhibition amongst analogs modified on sides, the following investigations were focused on analog **2** due to lack of consistency with solubility and stability issues with analog **12**. Dimethyl sulfoxide (DMSO) in which all these compounds were dissolved did not show any inhibition of luciferase activity.

**Scheme 2:**
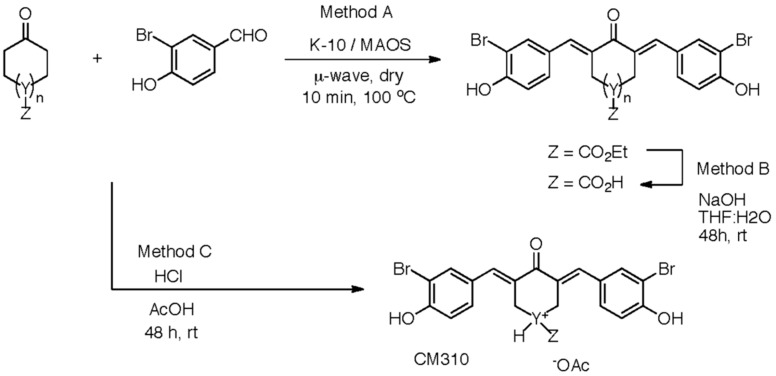
Synthetic methods were used to modify the central ring of *cy-C*_5_-curcumin to generate new analogs.

Scheme 2 shows the synthesis of the new analogs which were altered on the central ring of compound **1** (Figure 1). Table 2 lists the inhibitory effect of each compound, which was determined by a luciferase-based reporter assay that involved Dox-mediated activation of p53 response element. Supplementary Table S2 shows the raw luciferase data for calculating the percentage inhibition of each compound. The cyclopentanone **13**, methyl appended **14**, acid appended **16**, and hydroxy appended **17** analogs failed to show significant inhibitory activity. In addition, heteroatom analogs, pyranone **18**, and N-methylated piperidone **20** were not significantly more potent as compared to the other analogs in the list. Of the central ring-modified analogs, ester appended cyclohexenone **15** and piperidone salt **19** demonstrated the highest degree of inhibition. Taken together, these data suggest that inhibition of luciferase reporter activity could be due to the blocking of CBP-mediated p53K382ac, which is essential for activation of p53 transcription functions.

### 2.2. Biochemical Characterization of Potential Inhibitors of CBP HAT

#### 2.2.1. IC_50_ Determination to Identify the Most Potent Inhibitor of CBP HAT Activity

**Figure 2:**
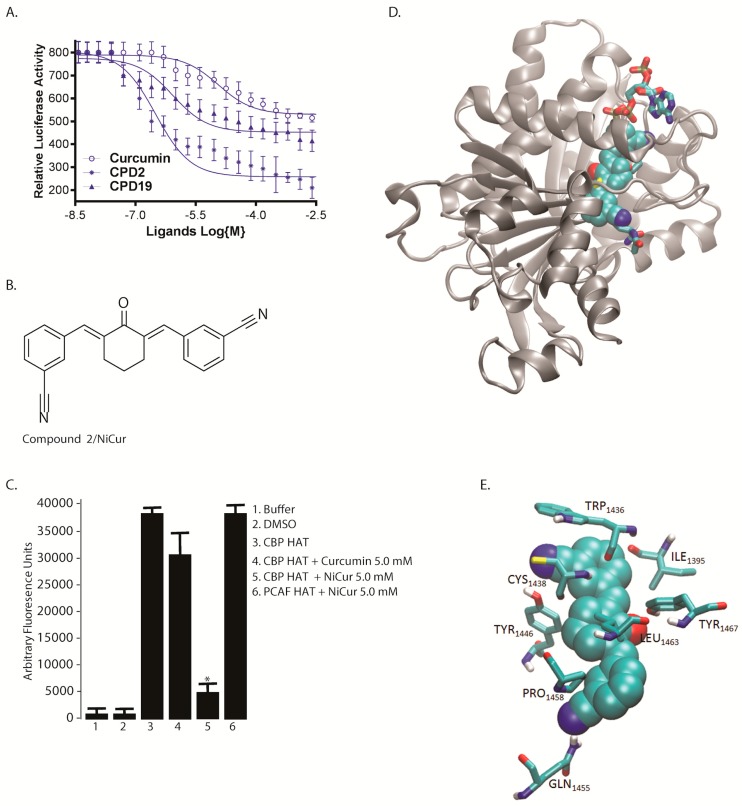
Biochemical characterization of compound **2** by cell-based assay and computational methods. (**A**) IC_50_ was determined by a Dox treated luciferase-driven p53 reporter assay using curcumin and compounds **2** (CPD 2) and 19 (CPD 19) in a dose-dependent manner. RLU stands for relative luciferase units. The IC_50_ values were calculated from three technical and biological repeats using the PRISM software; (**B**) Chemical structure of a *cy*-*C*_5_-curcuminoid-derived compound **2** that has been referred to as NiCur; (**C**) Relative inhibition of fluorescence-based in vitro CBP HAT activity in the presence of curcumin and NiCur at a concentration of 5.0 μM respectively. The data show mean ± SEM (*n* = *3*), with significance at *p* < 0.05 or *p <* 0.01 indicated by * and **, respectively; (**D**) NiCur (spheres) binding pose and the peptide inhibitor (sticks) in the active site of CBP/p300 are highlighted and the ribbon diagram depicts the rest of the protein; (**E**) The residues of CBP (sticks) interacting with NiCur (spheres).

The inhibitory capabilities of curcumin, compounds **2** and **19** were tested at increasing concentrations in Dox-treated U2OS cells, and later, determining their effects on p53 activation in a luciferase-based reporter assay. The data revealed that compound **2** was the most potent inhibitor with an IC_50_ value of 0.35 μM (Figure 2A). The IC_50_ values of curcumin and compound **19** were 10.0 and 1.0 μM, respectively. Taken together, given that compound **2** was at least 3-fold more potent than compound **19**, it could potentially inhibit CBP HAT-mediated acetylation leading to repression of the activation of p53. Figure 2B shows the chemical structure of the *cy*-*C*_5_-curcuminoid derived compound **2**, which is a nitrile-curcuminoid, named as NiCur.

#### 2.2.2. Effects of NiCur on the CBP HAT Activity

The respective potencies of NiCur and curcumin to inhibit CBP HAT activity were also tested in an in vitro HAT assay, which revealed that NiCur reduces the activity of CBP HAT by almost 80%. In comparison, curcumin inhibited CBP HAT activity by only about ~20% (Figure 2C). Notably, NiCur was not able to inhibit the HAT activity of another coactivator p300/CBP-associated factor (PCAF). DMSO and buffer in which the enzyme reactions were performed did not show any significant background activities. Together, the in vitro data indicate that NiCur has the ability to selectively inhibit CBP HAT activity.

#### 2.2.3. Molecular Basis of Interaction between CBP HAT Domain and NiCur

Structural analysis of CBP HAT domain revealed that the active site appears as a channel, which was lined on one side by β sheets and on the other side by α helices (Figure 2D). Notably, residues contributing to the channel formation were mostly polar. To further investigate whether NiCur binds to the active site of CBP HAT domain, in silico docking was performed. The model structure of the HAT/NiCur complex showed that NiCur extends across the active site within the channel (Figure 2D). Indeed, this orientation was comparable to the crystal structure of a bisubstrate inhibitor, Lys-CoA (PDB 3BIY; Figure 2D) [35]. An overlay of NiCur and Lys-CoA indicated that NiCur likely acts as a competitive inhibitor of CBP HAT at the active site. To explain the molecular basis of HAT/NiCur interaction, the complex structure showed that the side chains of Tyr 1446, Gln 1455, and Tyr 1467 form hydrogen bonds with NiCur, and simultaneously, the side chains of Pro 1458 and Leu 1463 make hydrophobic contacts with the ligand. Furthermore, the backbone carbonyl oxygen of Ile 1395, Trp 1436, and Cys 1438 are also in contact with the NiCur. The relative position of these residues with respect to NiCur is shown in Figure 2E. Taken together, these data confirm that NiCur is a more potent CBP HAT inhibitor than curcumin.

### 2.3. NiCur Modulates Transcription Functions of p53 during DNA Damage

**Figure 3:**
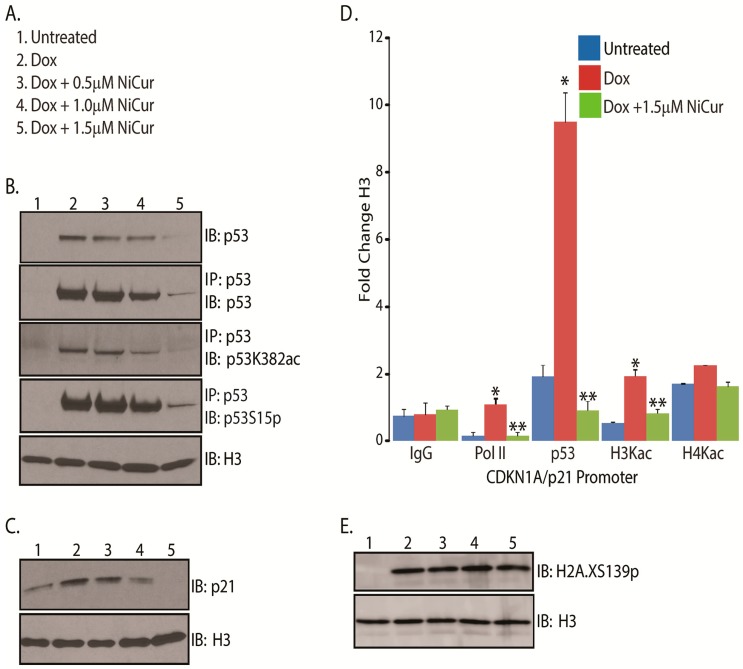
Dose-dependent inhibition of p53 in U2OS cells by NiCur. (**A**) Experimental conditions followed for the treatment of U2OS cells with Dox alone and together NiCur. The serial number denotes the experimental conditions with respect to the increasing concentrations of NiCur in the immunoblot panels; (**B**) IP and IB analysis showing expression levels of total p53, acetylated and phosphorylated p53 at lysine 382 and serine 15 in U2OS cells treated with conditions as described in (**A**). (**C**) IB analysis on the lysates of U2OS cells treated as per experimental conditions as described in (**A**) using antibodies to p21, and histone H3 (controls); (**D**) The fold change between the treatments of U2OS cells with Dox alone and together with NiCur that affected the enrichment of RNA Polymerase II, p53, H3Kac, and H4Kac on *CDKN1A/p21* promoter were expressed as the mean ± SEM (*n* = *3*), with significance at *p* < 0.05 or *p <* 0.01 indicated by * and ** respectively; (**E**) IB analysis using H2A.XS139p antibodies from the nuclear extracts of U2OS cells treated with conditions as described in (**A**).

Genotoxic stress induces p53S15p and p53K382ac, which are crucial for transcriptional functions of p53 [17,18]. Since the lysine 382 of tumor suppressor protein p53 is one of the major acetylation targets of CBP HAT, the effects of NiCur on the biochemical and transcriptional functions of p53 were investigated. To this end, U2OS cells were treated with the DNA damaging agent, Dox alone and together with increasing concentrations of NiCur at 0.50 μM, 1.0 μM, and 1.5 μM (Figure 3A). Subsequently, cellular extracts were subjected to immunoprecipitation (IP) using an anti-p53 antibody and immunoblot (IB) with anti-p53K382ac and p53S15p antibodies. The IB analysis following IP revealed that increasing concentrations of NiCur reduces the Dox-induced p53K382ac, p53S15p, and p53 levels in a dose-dependent manner (Figure 3B). Then, to verify whether the diminishing levels of p53K382ac and p53S15p were due to NiCur-mediated decline of p53 protein levels, the immunoblot signal of p53, p53K382ac, and p53S15p for a given condition as mentioned in Figure 3A were normalized with the respective signal of the control histone H3. The data revealed that downregulation of p53K382ac and p53S15p was indeed due to the attenuation of p53 expression by NiCur (Supplementary Table S3).

One of the major p53-directed cellular responses is the induction of cell cycle arrest, which is mediated by the increased expression of its downstream target *CDKN1A* or *p21* [17]. Therefore, to examine the effect of NiCur on *p21* activation, U2OS cells were treated with Dox alone and together with NiCur, according to the conditions as described in Figure 3A. IB analysis revealed that increasing concentrations of NiCur inhibits the expression of p53, which negatively affects the induction of p21 protein even after treatment with Dox (Figure 3C).

Stress-induced activation of *p21* expression requires the recruitment of activated p53 acetylated on lysine 382 and RNA Polymerase II on the promoter of the *p21*, which could be accompanied by acetylation of histone proteins H3 and H4 within the chromatin. To address this question, we next investigated whether NiCur modulated the recruitment of p53 and RNA Polymerase II as well as altered the levels of acetylation on histone H3 (H3Kac) and histone H4 (H4Kac) on the *p21* promoter. Subsequently, ChIP-qPCR data revealed a 10-fold increase in the levels of p53, 2-fold increase in the levels of H3Kac as well as 1.5-fold enrichment of RNA polymerase II on the *p21* promoter after treatment with Dox. This enrichment of p53 on *p21* promoter was downregulated significantly by NiCur (Figure 3D). Besides, the levels of H3Kac, which almost doubled after Dox treatment, declined due to the presence of NiCur (Figure 3D). Comparatively, NiCur was more effective in diminishing the levels of H3Kac than H4Kac. Further, decline in the enrichment of RNA Polymerase II was noted, which indicates that CBP acts as a functional bridge between p53 and RNA Polymerase II and acetylated chromatin [36].

Finally, to test whether NiCur modulates upstream signals of DNA damage pathway, phosphorylation at serine 139 on histone H2A.X (H2A.XS139p) was investigated in U2OS cells after Dox treatment as described in Figure 3A. Besides, H2A.XS139p serves as one of the salient marks for sensing DNA damage, which later culminates in activation of p53 [37]. Subsequently, the data after performing IB on the nuclear extracts of U2OS cells reveal that NiCur did not affect the levels of H2A.XS139p induced by Dox (Figure 3E). Taken together, these results suggest that inhibition of p53K382ac is due to the downregulation of p53 activation and expression, which subsequently leads to the downregulation of *p21* expression without affecting the levels of H2A.XS139p.

### 2.4. NiCur Dynamically Reprograms the Acetylation Marks on the Chromatin Landscape

**Figure 4:**
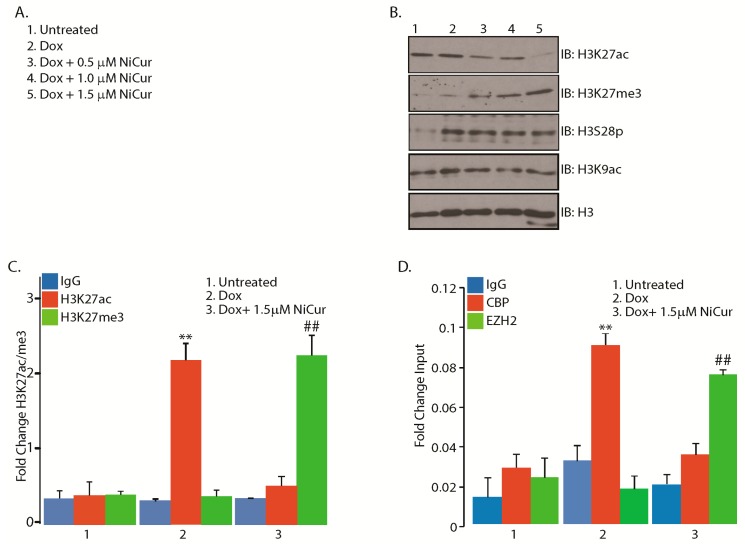
NiCur reprograms chromatin landscape from acetylation, H3K27ac, to *tri*methylation on lysine 27 of histone H3, H3K27me3. (**A**) Experimental conditions followed for the treatment of U2OS cells with Dox alone and together-with NiCur; (**B**) The nuclear extracts from U2OS cells treated with Dox alone and together-with NiCur were subjected to IB, which revealed that without affecting the levels of H3K9ac and H3S28p, downregulation of H3K27ac in the presence of NiCur led to an increase in the level of H3K27me3 on the chromatin; (**C**) ChIP-qPCR assay on the promoter of *CDKN1A/p21* promoter demonstrates that treatment of U2OS cells by Dox results in ~2-fold increase acetylation on H3K27 site, which is reversed by NiCur that leads to a ~2-fold increase in methylation; (**D**) Increased occupancy of CBP by ~3-fold on *CDKN1A/p21* promoter was reversed by the occupation of EZH2 after treatment of U2OS cells with NiCur. The fold changes upon the treatment of U2OS cells with Dox alone and together-with NiCur that lead to enrichment of H3K27ac/CBP versus H3K27me3/EZH2 were expressed as the mean ± SEM (*n* = *3*), with significance at *p* < 0.05 or *p <* 0.01 as indicated by ** and ##, respectively.

Besides p53K382ac, H3K27ac mediated by CBP serves as a mark for gene activation [23]. However, H3K27me3 mediated by EZH2 leads to gene silencing [24]. Thus, to examine the effect of NiCur on the levels of H3K27ac, IB analysis was performed on the nuclear extracts from U2OS cells, which were treated with conditions as described in Figure 4A. The data revealed that NiCur inhibits H3K27ac. However, unlike p53, where blocking of p53K382ac reduced the expression as well as activation of p53, the concentration-dependent decline in the levels of H3K27ac was concomitant with an increase in H3K27me3 (Figure 4B). Importantly, the H3K9 site, which is acetylated by another HAT containing coactivator, PCAF, remained unaffected. The induction of phosphorylation at the H3S28 (H3S28p) site by Dox remains unaltered by NiCur treatment (Figure 4B).

To confirm whether enhancement of H3K27me3 mark on the chromatin occurred on the *p21* promoter, ChIP-qPCR was performed with the chromatin of U2OS cells after treatment with Dox in presence and absence of NiCur at 1.5 μM. The results show that Dox alone was able to enhance the level of H3K27ac 2-fold (Figure 4C). However, NiCur reduces the level of H3K27ac, which was concomitant with the enhancement of H3K27me3 mark on *p21* promoter. These findings suggest the existence of dynamic and bivalent histone marks (H3K27me3 versus H3K27ac) on the promoter of *p21* that could be modulated by Dox alone or by a CBP HAT inhibitor, such as NiCur.

Next, whether NiCur-mediated downregulation of H3K27ac, which was concomitant with upregulation of H3K27me3, modulates the occupation of CBP versus EZH2 on the *p21* promoter was investigated. To this end, ChIP-qPCR was performed with anti-CBP and anti-EZH2 antibodies as well as IgG included as a control. The ChIP-qPCR data revealed that treatment with Dox enhanced the enrichment of CBP, in contrast to the treatment with NiCur, which led to the enhancement of EZH2 occupation on the *p21* promoter (Figure 4D). Collectively, these results suggest that NiCur reprograms the chromatin landscape globally as well as on the *p21* promoter by diminishing the levels of CBP as well as H3K27ac and by simultaneously increasing the presence of EZH2 as well as H3K27me3.

### 2.5. Cellular Effects of NiCur Treatment on Normal Rat Intestinal Epithelial Cells

**Figure 5:**
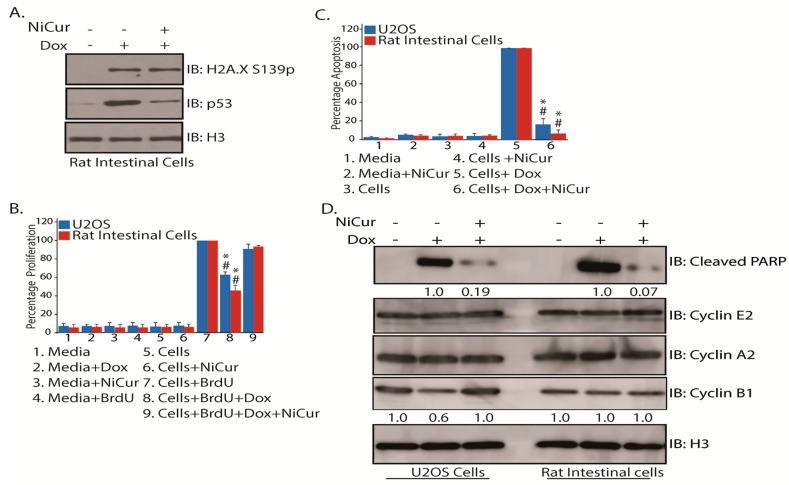
NiCur inhibits cellular proliferation and apoptosis in U2OS and normal rat gastrointestinal epithelial cells. (**A**) NiCur downregulates Dox-mediated p53 activation in normal rat intestinal cells without affecting the levels of H2A.X S139p; (**B**) Incorporation of BrdU after treatment of U2OS and normal rat gastrointestinal epithelial cells with Dox in presence and in absence of 1.5 μM of NiCur was quantitated by measuring optical density (OD) at 450 nm. The OD obtained was normalized to 100% for BrdU-treated U2OS and normal rat intestinal epithelial cells, and then, all the conditions (1–9) were calculated as 100 *OD/OD of BrdU-treated cells; (**C**) The intracellular levels of Cleaved Caspase 3 in U2OS and normal rat gastrointestinal epithelial cells after treatment with Dox in presence and in absence of 1.5 μM of NiCur was measured at OD of 450 nm. The OD was normalized to 100% for Dox-treated U2OS and normal rat intestinal epithelial cells and then, all the conditions (1–6) were calculated as 100 *OD/OD of Dox-treated cells. All experiments were performed in triplicates of technical and biological repeats. The experimental values are expressed as the mean ± SEM (*n* = *3*), with significance at *p* < 0.05 or *p <* 0.01 indicated by * and #, respectively. (**D**) NiCur downregulates p53-mediated apoptosis in U2OS and normal rat intestinal epithelial cells as demonstrated by the attenuation of cleavage of PARP without affecting the levels of Cyclins A2 and E2. The signals for the levels of Cleaved PARP after Dox treatment in U2OS and gastrointestinal epithelial cells were determined by densitometry and then normalized to 1 and later the effect of NiCur was calculated respectively. Besides, p53-mediated downregulation of the expression of Cyclin B1 in U2OS cells was restored by 1.5 μM of NiCur treatment.

One of the major side-effects from anticancer therapies is the activation of p53-directed loss of normal cells by apoptosis, which results in the physiological exacerbation of cancer patients. To investigate whether NiCur inhibits p53-activation, normal rat gastrointestinal epithelial cells were subjected to Dox treatment in presence and absence of NiCur. Then, IB analysis on the nuclear extracts from these cells showed that though Dox-induced DNA damage was marked by increase in the levels of H2A.XS139p, it remained unaffected by the treatment of NiCur. However, NiCur was able to reduce the levels of p53 in these cells (Figure 5A).

As NiCur inhibited Dox-mediated activation of p53, it was vital to confirm the effects of NiCur on the cellular proliferation and viability of U2OS and normal rat intestinal epithelial cells. To address this question, U2OS and normal gastrointestinal epithelial cells were incubated with 5-bromo-2-deoxyUridine (BrdU) as well as treated with Dox in presence and absence of NiCur. The data, as shown in Figure 5B, demonstrate that Dox treatment reduces the BrdU incorporation by approximately 37% in U2OS and about 54% in normal rat gastrointestinal epithelial cells. However, the cellular proliferation was restored when these cells were treated with NiCur in presence of Dox.

Reduction in the cellular proliferation in Dox-treated cells could be due to either p53-mediated growth arrest or apoptosis. For testing this conjecture, cellular levels of cleaved Caspase 3, which is one of key determinants for triggering apoptosis, was determined by ELISA. The data, as shown in Figure 5C, reveal that NiCur inhibits apoptosis in U2OS cells by approximately 88% and in rat gastrointestinal epithelial cells by 95%. These results indicate that NiCur inhibits p53-mediated apoptosis in U2OS and normal rat gastrointestinal epithelial cells by blocking the CBP HAT function.

Attenuation of p53-mediated apoptosis by inhibiting Caspase 3 activities suggested further examination for the effect of NiCur on cell cycle. To address this point, the levels of cleaved PARP, a marker for active apoptosis and Cyclins E2, A2, and B1, which regulate the transition of cell cycle events, G1, S, and G2M, were determined. Subsequently, after treatment with Dox alone and together-with NiCur, the IB analysis revealed that Dox-mediated activation of p53 led to the induction of PARP cleavage in both U2OS and normal rat gastrointestinal epithelial cells. However, induction of cleavage of PARP was significantly inhibited by NiCur in both cell types. Notably, the induction of PARP cleavage in normal rat gastrointestinal epithelial cells was observed to be almost twice more than in U2OS cells (Figure 5D). Besides, inhibition of PARP cleavage by NiCur treatment was relatively more dramatic in normal rat gastrointestinal epithelial cells as compared to U2OS cells (Figure 5D). Then, due to Dox-mediated activation of p53, Cyclin B that mediates G2/M transition was reduced approximately by 40% in the U2OS cells as compared to the normal rat gastrointestinal epithelial cells (Figure 5D). However, NiCur treatment restored the levels of Cyclin B similar to the untreated cells. Finally, the levels of Cyclins E2 and A2, which mediate transition of G1/S and S phases, remained largely unaffected in both U2OS and normal rat gastrointestinal epithelial cells (Figure 5D). Collectively, although apoptosis is devastating to the normal cells, these results indicate that it could be blocked by inhibiting the function of CBP HAT. 

In summary, the present study demonstrates the synthesis and biochemical as well as cellular effects of one of the most potent CBP HAT inhibitors, NiCur. The in vitro enzyme assay with CBP HAT shows that NiCur inhibits the CBP HAT activity, but not that of PCAF HAT. Subsequently, computational modeling confirms that NiCur docked into the active site pocket of CBP HAT. The biochemical analysis reveals that NiCur attenuated the transcriptional activation of p53 by downregulating its expression. Further, NiCur facilitates the reprogramming of the chromatin landscape by switching from H3K27ac to H3K27me3. On the *CDKN1A*/*p21* promoter, NiCur blocks the recruitment of p53, RNA Polymerase II, and is accompanied by the reduction of acetylation levels on histone H3. Finally, NiCur mitigates p53-mediated apoptosis in normal rat gastrointestinal epithelial cells.

## 3. Discussion

Lysine acetylation is essential for regulating the biochemical activities of transcription factors as well as orchestrating the downstream genes, which control key cellular processes during normal and disease situations [7,8]. However, acetylation-specific function of the master coactivator, CBP, remains unclear mainly due to its large size and multi-domain constitution [10]. The process of gene regulation is orchestrated by well-organized domains that constitute CBP, namely: (i) three cysteine-rich modules (CH1 to CH3) are involved in zinc binding and protein–protein interactions; (ii) a HAT domain in the center of the protein that mediates lysine acetylation; (iii) a BRD that binds to a acetylated-lysine moiety; (iv) a PHD motif, also involved in protein–protein interactions; (v) two transactivation domains located at either end of CBP; and (vi) multiple specific interaction domains for different transcription factors, such as the KIX domain that mediates the interaction of CBP with phosphorylated CREB at Ser133 [36]. Recruitment of either a BRD-containing protein or a histone deacetylase to the acetylated-lysine moiety could lead to either gene activation or repression, complicating our understanding of the downstream effect of CBP-mediated acetylation [38]. Furthermore, in a given biological context, overexpression and gene knockout strategies are limited in revealing the functional significance of a specific domain in regulating the overall function of the protein, like CBP. Together, a small molecule that will not perturb expression of endogenous CBP, but only modulate the function of a specific domain, has a greater potential to precisely reveal the biological significance of CBP epigenetic functions.

Previous studies have established that CBP HAT-mediated acetylation controls the transcriptional activity of p53 for regulating the function of target genes that modulate cellular response upon multitude of stress [16,18,39]. One of the severe consequences of anticancer therapies is the activation of p53-directed apoptosis, which is pivotal for controlling tumor growth. However, it attains a pathological dimension by inducing undesired cell death in normal tissues [40]. The present study demonstrates the development and biochemical characterization of a chemical modulator, NiCur that blocks CBP HAT activity. A *cy*-*C*_5_-curcumin analog, **1**, which was selected as a starting point, because of its nutritional significance, was much less potent. NiCur with an IC_50_ of 0.35 μM, which was the most potent analog demonstrated cellular effects at 0.50 to 1.5 μM that could be due to the overexpression of luciferase protein in contrast to the level of endogenous CBP. Though NiCur and compounds **12** and **19** showed similar inhibitory potential at 0.50 μM, NiCur demonstrated consistency in concentration-dependent inhibition of p53 reporter assay. The in vitro HAT assay revealed that compound NiCur could inhibit the HAT activity of CBP, but not of PCAF. Computational modeling shows that NiCur docked into the active site of CBP HAT. Further, in contrast to negatively affecting the activation of p53 protein, NiCur, dynamically orchestrated the level of H3K27ac, which led to the increase in the level of H3K27me3 and the occupation of histone methyltransferase, EZH2. Possibly, the trend showing decline in the expression level of p53 at 1.0 and 1.5 μM concentrations could be due to an increase in the level of H3K27me3 on the *p53* promoter. Data indicated that increase in the level of H3K27me3 was independent of DNA damage. Notably, reduction in the levels of H3Kac was associated with diminished levels of RNA Polymerase II on the promoter of *CDKN1A/p21*. These data support that CBP acts as a functional transcriptional bridge between p53, RNA Polymerase II, and chromatin [41]. Also, induction in the level of phosphorylation at serine 28 on histone H3 due to Dox remained unaffected by NiCur treatment.

Clearly, NiCur inhibited Dox-mediated activation of p53 in osteosarcoma U2OS cell lines, which served as an appropriate cellular model to characterize the biological activities of NiCur. However, the major goal of this research investigation was to identify and test the biochemical ability of the most potent CBP HAT inhibitor in blocking p53-directed apoptosis in normal cells. The rationale for testing NiCur in rat gastrointestinal epithelial cells stemmed from previous data in murine models, which demonstrated that the gastrointestinal tract is one of the major targets showing pathology due to anticancer therapies [5,40]. Like Caspase 3 activity, NiCur inhibits p53-directed cleavage of PARP in U2OS and rat gastrointestinal epithelial cells. Additionally, it is known that the DNA damage-mediated activation of p53 reduces the expression of Cyclin B1, which was overcome by NiCur. Previous reports have established that tumor suppressor p53-mediated downregulation of Cyclin B1 is an indicative of G2M arrest [41]. Possibly, like p53-mediated repression of Cyclin B1, expression of Cyclin A2 could also be reduced at a later time-point [42]. Further, results showing the cellular effects of NiCur fully concur with our hypothesis that pharmacological blocking of CBP HAT activity can downregulate p53 functions in normal cells. Taken together, treatment with NiCur demonstrates that acetylation could be essential for regulating the activation of p53 protein. A future goal will be to develop HAT inhibitors with a lower nanomolar affinity that are active in primary cells and xenograft models of human cancers.

Published studies suggest that curcumin has a poor bioavailability, which is indicated by its low serum presence, rapid metabolism, and restricted tissue distribution [43]. The relatively higher number of rotatable bonds (8) provides curcumin with the plasticity to target multiple binding pockets leading to off target effects. Also, the large polar surface area indicates the ability of curcumin to form electrostatic interactions with its corresponding target. Notably, compared to curcumin, NiCur, has smaller molecular weight with only 2 rotatable bonds, smaller polar surface area, and is still below the Lipinski limit of log P value. The small number of rotatable bonds ensures that it will only bind to very few targets and helps the binding by reducing the entropy penalty upon binding that in turn can compensate for the reduction in polar surface area, ensuring binding strength comparable to that of curcumin (data not shown). Compounds **12** and **19** lack consistent biochemical activity in concentration-dependent inhibition assay for p53 activation, thereby, indicating potential toxicity, lack of solubility, or cell type specific issues. However, given that the transcriptional coactivator CBP is central to the activation of androgen receptor and NF-κB, pilot studies will be performed to test these compounds in the cellular models of prostate cancer and inflammation [44,45]. One of the caveats for inhibiting apoptosis could be the emergence of chemoresistance that would promote growth in tumors with wild type *p53*. However, *p53* is mutated in many cancers, and regardless; a mutant p53 protein undergoes acetylation, which also assists in tumor growth [46]. Most likely, downregulating a mutant *p53* by a CBP HAT inhibitor could restrict the tumor growth. Furthermore, p53 supports the pathogenesis of neurodegenerative diseases, which further expands the value of a CBP HAT inhibitor [47]. Finally, a published report demonstrates that a pharmacological blocker of p53, CHIR99021, inhibits apoptosis and improves Lgr51 cell survival and crypt regeneration, thereby preventing radiation-induced pathology in cellular and mice models [3].

The H3K27 site undergoes acetylation as well as mono, di or *tri*methylation but in a mutually exclusive manner. Site-specific acetylation of H3K27 is solely mediated by CBP, whereas *tri*methylation of H3K27 site is solely mediated by EZH2 [24]. Consequently, H3K27ac serves as a mark for gene activation and H3K27me3 serves as a mark for gene repression [24]. Data emerging from the present study suggest that NiCur could modulate the gene regulatory switch for reprogramming chromatin landscape. Given that CBP acetylates several sites on histone H3 and within the nucleosomes, including H3K18, H3K23, H3K27, H3K56, H2AK5, H2BK2, and H2BK5, it is likely that global histone acetylation will be reduced by NiCur [47]. Comparatively, a small molecule has the advantage of being able to reveal domain-specific function of an endogenous and a large protein, like CBP (265kDa), over RNAi, gene knockout and overexpression strategies while dissecting molecular mechanisms. In future investigations, NiCur could be used as a mechanistic tool to dissect differential recruitment and molecular interplay of proteins involved in nucleosomal remodeling during transition of chromatin landscape from H3K27ac to H3K27me3. To summarize, a selective small molecule could be used for performing mechanistic studies without affecting the expression of target proteins.

Bisubstrate inhibitors of CBP HAT function lack cell permeability [48]. Garcinol inhibits histone acetylation at 5.0 μM in cells treated with the histone deacetylase inhibitor, TSA [49]. Similarly, a natural product inhibits histone acetylation at 8.5 μM. Our data demonstrate that NiCur inhibits HAT functions of CBP in lower micromolar range. The HAT/NiCur complex structure can be used to design the mutants of CBP HAT active site to verify the putative binding pose. Furthermore, it can also be used to predict its selectivity by comparing the binding sites of possible off-target proteins with that of CBP and to design potent analogs of NiCur to enhance its selectivity for other HATs. CBP and p300 share at least 60% of the residues, which are identical and 70% of their residues are similar. In particular, HAT domains between CBP and p300 share 86% of the homology [36]. Given that HAT domains of p300 and CBP are highly homologous it is possible that p300 could also be affected by compound NiCur and the effects of p300 HAT suppression may not necessarily be the same as CBP HAT. Together, unraveling the underlining biochemical complexity, which guarantees selectivity to CBP with respect to other cellular HATs by developing more selective anti-CBP HAT small molecules, will be the future goal.

## 4. Conclusions

This study lays the foundation for developing high affinity CBP HAT modulators that could serve as a valuable mechanistic tool for transforming cellular fate by reprogramming the epigenetic landscape. We expect that tapping into the chemical biology of high affinity ligands for enzymes, which intrinsically modulate p53 and chromatin functions, will facilitate dissection of complex regulation of p53 functions during anticancer therapies in normal cells. Besides ameliorating the side-effects of anticancer therapies, pharmacological modulation of p53 will have translational significance in cancers with mutant *p53* and many disease conditions, including neurodegenerative diseases. 

## 5. Materials and Methods

### 5.1. Synthetic Methods for Designing the Two-Prong Library of Potential CBP HAT Inhibitors

Synthetic Method A, a dry-phase Claisen-Schmidt condensation, was used to access *cy*-*C*_5_-curcumin analogs with various sides (Scheme 1). Method A was employed for bromo-hydroxy (1), nitrile (2), bromo-methoxy (3), alkyl (4−5), alkyl-hydroxy (6), hydroxyl (7), halo (8−11), and pyridinyl (12) functionalized benzaldehydes with the cyclohexaneone central ring. Montmorillonite K-10 clay, a solid-acid catalyst, was utilized with MAOS [50,51]. Method A with heterocyclic *m*-pyridinyl benzaldehyde was successful, but *o*- and *p*-pyridinyl benzaldehydes were incompatible under the acid mediated conditions [52,53]. Method A provided pure crystalline products for testing.

Synthetic methods A, B, and C were used to access *cy*-*C*_5_-curcumin analogs with different central rings (Scheme 2). Method A was employed for cyclohexanones (1, 14−15, 17), cyclopentanone (13), dihydro-2*H*-pyran-4-one (18), and *N*-methylpiperid-4-one (20) with 3-bromo-4-hydroxybenzaldehyde sides. Hydroxy, protected with *tert*-butyldimethylsilyl (TBDMS), cyclohexanone underwent condensation with concomitant TBDMS deprotection for the hydroxy pendant analog (17). Ethyl 4-oxocyclohexanecarboxylate underwent condensation to provide the ester pendant analog (15), which was hydrolyzed to the acid pendant analog (16, Method B). Method C, conducted in solution, used HCl in acetic acid with piperidin-4-one hydrochloride hydrate and aldehyde. Overall the two-prong approach provided diverse, crystalline products for biological testing.

### 5.2. Chemical Synthesis and General Information

All reagents were obtained from commercial suppliers and used without further purification. ^1^H-NMR was collected at resonance frequency 600 MHz with chemical shifts reported in ppm from tetramethylsilane (TMS) (Bruker, Billerica, MA, USA). Microwave irradiation was applied to sealed microwave vials in a single mode Biotage^®^-Initiator cavity (Biotage, Charlotte, NC, USA), producing continuous irradiation at 2.45 GHz. Automated flash chromatography was conducted on prepacked KP-Sil™ columns (Biotage) attached to a Biotage^®^-Isolera Four instrument, monitoring UV Trace at 254 nm, with solvent systems A = dichloromethane/hexanes; B = dichloromethane/MeOH; or C = hexanes/ethyl acetate. LC-MS (ESI) analysis was performed on an Agilent^®^ 1200 HPLC (Agilent, Santa Clara, CA, USA) equipped with a Zorbax^®^ 300SB-C18 column (Agilent, Santa Clara, CA, USA) held at 45 °C, and a G1969A API-TOF (Agilent) in positive mode. Purity of all the compounds was estimated at >95% by the diode array detector (DAD) trace using the following Liquid Chromatography (LC) method: solvent system (A)/(B) with 0.1% buffer = H_2_O/acetonitrile with formic acid, flow rate = 0.4 mL/min, and timed percentage of solvent B = 0−1 min (1%), gradient 1−4 min (1–99%), 4−8 min (99%). HRMS results were obtained from the Mass Spectrometer Core Facility at Columbia University using a HX-110 double focusing high resolution MS JEOL Ltd. Tokyo, Japan, with 10 KV Accel. Volt, FAB ionization Xe, 3 KV collision energy.

#### 5.2.1. Claisen-Schmidt Condensation General Procedure

Method A:

Aldehyde (2 eq) and Montmorillonite K-10 (0.800 g) were dissolved in methanol (1 mL) followed by evaporation to dryness on the rotavap. The aldehyde dispersion was added to a microwave vessel followed by the ketone (1.0 mmol, or 10.0 mmol) and sealed with a septum. The sample was irradiated at 100 °C for 5 min. The crude material was added to a filter funnel and washed 3 times with alternating portions of methanol (5 mL) and dichloromethane (5 mL). The solvents were removed under reduced pressure and the crude products were purified by flash chromatography or recrystallization, or both techniques.

*(2E,6E)-2,6-bis(3-Bromo-4-hydroxybenzylidene)cyclohexanone* (**1**) [54]: Yellow microneedles (30 mg, 7% yield) from dichloromethane/hexanes. Solvent system A, elution at 50% dichloromethane. ^1^H-NMR (MeOD) δ 1.81–1.82 (m, 2H), 2.91(s, 4H), 6.94(d, *J* = 8.4 Hz, 2H), 7.36 (d, *J* = 8.2 Hz, 2H), 7.58–7.65 (m, 4H). LCMS *t* = 6.2 min, *m*/*z* Calcd. for C_20_H_17_Br_2_O_3_; C_20_H_16_Br_2_NaO_3_; C_40_H_32_Br_4_NaO_6_ 464.95, 462.95, 466.95; 486.93; 950.88 [M + H]^+^; [M + Na]^+^; [2M + Na]^+^, Found 464.94, 462.94, 466.94; 486.92; 950.93.

*3,3′-((1E,1′E)-(2-Oxocyclohexane-1,3-diylidene)bis(methanylylidene))dibenzonitrile* (**2**): *nitrile-curcuminoid/NiCur)* [55]: Yellow microcrystals (38 mg, 12% yield) from dichloromethane/hexanes. Solvent system B, elution at 100% dichloromethane. ^1^H-NMR (CDCl_3_) δ 1.82–1.85 (m, 2H), 2.91 (*t*, *J* = 5.2 Hz, 4H), 7.54 (*t*, *J* = 7.7 Hz, 4H), 7.63–7.67 (m, 4H), 7.72 (*s*, 2H). LCMS *t* = 6.3 min, *m*/*z* Calcd. for C_22_H_17_N_2_O; C_22_H_16_N_2_NaO; C_44_H_32_N_4_NaO_2_ 325.13, 326.14; 347.12; 671.24 [M + H]^+^; [M + Na]^+^; [2M + Na]^+^, Found 325.16, 326.14; 347.14; 671.25.

*(2E,6E)-2,6-bis(3-Bromo-4-methoxybenzylidene)cyclohexanone* (**3**): Yellow sheets (44 mg, 9% yield) from dichloromethane/hexanes. Solvent system A, elution at 75% dichloromethane. 1H-NMR (DMSO-*d*_6_) δ 1.73 (*t*, *J* = 5.5 Hz, 2H), 2.86–2.88 (m, 4H), 3.90 (s, 6H), 7.20 (d, *J* = 8.6 Hz, 2H), 7.54 (s, 2H), 7.57 (d, *J* = 8.4 Hz, 2H), 7.78 (s, 2H). LC *t* = 7.0 min. HRMS *m*/*z* Calcd. for C_22_H_21_Br_2_O_3_ 490.9857 [M + H]^+^, Found 490.9855.

*(2E,6E)-2,6-bis(2-Methylbenzylidene)cyclohexanone* (**4**) [56]: Yellow sheets (25 mg, 8% yield) from dichloromethane/hexanes. Solvent system A, elution at 50% dichloromethane. ^1^H-NMR (CDCl_3_) δ 1.71 (*t*, *J* = 5.5 Hz, 2H), 2.35 (s, 6H), 2.77 (*t*, *J* = 5.8 Hz, 4H), 7.21–7.26 (m, 8H), 7.90 (s, 2H). LCMS *t* = 7.1 min, *m*/*z* Calcd. for C_22_H_23_O; C_22_H_22_NaO; C_44_H_44_NaO_2_ 303.17, 304.18; 325.16; 627.32 [M + H]^+^; [M + Na]^+^; [2M + Na]^+^, Found 303.21, 304.21; 325.19; 627.32.

*(2E,6E)-2,6-bis(3,5-Dimethylbenzylidene)cyclohexanone* (**5**): Yellow needles (12 mg, 4% yield) from EtOH. Solvent system A, elution at 50% dichloromethane. ^1^H-NMR (CDCl_3_) δ 1.77–1.78 (m, 2H), 2.35 (s, 12H), 2.91–2.93 (m, 4H), 6.98 (s, 2H), 7.08 (s, 4H), 7.74 (s, 2H). LC *t* = 7.3 min. HRMS *m*/*z* Calcd. for C_24_H_27_O 331.2062 [M + H]^+^, Found 331.2054.

*(2E,6E)-2,6-bis(4-Hydroxy-3,5-dimethylbenzylidene)cyclohexanone* (**6**) [57]: Yellow sheets (35 mg, 10% yield) from chloroform. Solvent system B, elution at 100% dichloromethane. ^1^H-NMR (MeOD) δ 1.78–1.80 (m, 2H), 2.24 (s, 12H), 2.92–2.94 (m, 4H), 7.14 (s, 4H), 7.61 (s, 2H). LCMS *t* = 6.1 min, *m*/*z* Calcd. for C_24_H_27_O_3_; C_24_H_26_NaO_3_; C_48_H_52_NaO_6_ 363.20, 364.20; 385.18; 747.37 [M + H]^+^; [M + Na]^+^; [2M + Na]^+^, Found 363.21, 364.21; 385.18; 747.38.

*(2E,6E)-2,6-bis(4-Hydroxybenzylidene)cyclohexanone* (**7**) [57]: Green microneedles (15 mg, 5% yield) from dichloromethane/MeOH. Solvent system B, elution at 5% MeOH. ^1^H-NMR (MeOD) δ 1.80 (*t*, *J* = 5.8 Hz, 2H), 2.93 (*t*, *J* = 5.1 Hz, 4H), 6.84 (d, *J* = 8.5 Hz, 4H), 7.40 (d, *J* = 8.5 Hz, 6H), 7.67 (s, 2H). LCMS *t* = 5.8 min, *m*/*z* Calcd. for C_20_H_19_O_3_; C_20_H_18_NaO_3_; C_40_H_36_NaO_6_ 307.13, 308.14; 329.11; 635.24 [M + H]^+^; [M + Na]^+^; [2M + Na]^+^, Found 307.17, 308.17; 329.14; 635.24.

*(2E,6E)-2,6-bis(2-Fluorobenzylidene)cyclohexanone* (**8**) [58]: Yellow microcrystals (20 mg, 6% yield) from hexanes. Solvent system A, elution at 50% dichloromethane. ^1^H-NMR (CDCl_3_) δ 1.77 (*t*, *J* = 5.8 Hz, 2H), 2.80–2.82 (m, 4H), 7.09–7.18 (m, 4H), 7.31–7.39 (m, 4H), 7.83 (s, 2H). LCMS *t* = 6.8 min, *m*/*z* Calcd. for C_20_H_17_F_2_O; C_20_H_16_F_2_NaO; C_40_H_32_F_4_NaO_2_ 311.12, 312.13; 333.11; 643.22 [M + H]^+^; [M + Na]^+^; [2M + Na]^+^, Found 311.16, 312.16; 333.14; 643.23.

*(2E,6E)-2,6-bis(2-Bromobenzylidene)cyclohexanone* (**9**) [58]: Yellow microcrystals (22 mg, 5% yield) from hexanes. Solvent system A, elution at 50% dichloromethane. ^1^H-NMR (CDCl_3_) δ 1.76 (*t*, *J* = 5.8 Hz, 2H), 2.76 (s, 4H), 7.20 (*t*, *J* = 6.4 Hz, 2H), 7.31–7.33 (m, 4H), 7.64 (d, *J* = 8.0 Hz, 2H), 7.85 (s, 2H). LCMS *t* = 7.1 min, *m*/*z* Calcd. for C_20_H_17_Br_2_O; C_20_H_16_Br_2_NaO; C_40_H_32_Br_4_NaO_2_ 432.96, 430.96, 434.96; 454.95; 886.89 [M + H]^+^; [M + Na]^+^; [2M + Na]^+^, Found 432.96, 430.96, 434.96; 454.94; 886.96.

*(2E,6E)-2,6-bis(3-Bromobenzylidene)cyclohexanone* (**10**) [59] Yellow microcrystals (50 mg, 12% yield) from hexanes. Solvent system A, elution at 50% dichloromethane. ^1^H-NMR (CDCl_3_) δ 1.81 (*t*, *J* = 6.0 Hz, 2H), 2.90 (*t*, *J* = 5.3 Hz, 4H), 7.26–7.30 (m, 2H), 7.37 (d, *J* = 7.6 Hz, 2H), 7.47 (d, *J* = 7.8 Hz, 2H), 7.59 (s, 2H), 7.70 (s, 2H). LCMS *t* = 7.4 min, *m*/*z* Calcd. for C_20_H_17_Br_2_O; C_20_H_16_Br_2_NaO; C_40_H_32_Br_4_NaO_2_ 432.96, 430.96, 434.96; 454.95; 886.89 [M + H]^+^; [M + Na]^+^; [2M + Na]^+^, Found 432.95, 430.95, 434.94; 454.93; 886.93.

*(2E,6E)-2,6-bis(4-Bromobenzylidene)cyclohexanone* (**11**) [60]: Yellow microneedles (22 mg, 5% yield) from hexanes. Solvent system A, elution at 50% dichloromethane. ^1^H-NMR (DMSO-*d*_6_) δ 1.72 (*t*, *J* = 5.7 Hz, 2H), 2.87 (*t*, *J* = 5.1 Hz, 4H), 7.50 (d, *J* = 8.3 Hz, 4H), 7.57 (s, 2H), 7.65 (d, *J* = 8.3 Hz, 4H). LCMS *t* = 7.3 min, *m*/*z* Calcd. for C_20_H_17_Br_2_O; C_20_H_16_Br_2_NaO; C_40_H_32_Br_4_NaO_2_ 432.96, 430.96, 434.96; 454.95; 886.89 [M + H]^+^; [M + Na]^+^; [2M + Na]^+^, Found 432.96, 430.96, 434.96; 454.94; 886.96.

*(2E,6E)-2,6-bis(Pyridin-3-ylmethylene)cyclohexanone* (**12**): Yellow microneedles (20 mg, 7% yield) from dichloromethane/hexanes. Solvent system B, elution at 5% MeOH. ^1^H-NMR (CDCl_3_) δ 1.82–1.87 (m, 2H), 2.95 (*t*, *J* = 5.6 Hz, 4H), 7.34–7.37 (m, 2H), 7.76–7.78 (m, 4H), 8.58 (d, *J* = 4.5 Hz, 2H), 8.73 (s, 2H). LCMS *t* = 5.0 min, *m*/*z* Calcd. for C_18_H_17_N_2_O; C_18_H_16_N_2_NaO; C_36_H_32_N_4_NaO_2_ 277.13, 278.14; 299.11; 575.24 [M + H]^+^; [M + Na]^+^; [2M + Na]^+^, Found 277.18, 278.18; 299.15; 575.22.

*(2E,5E)-2,5-bis(3-Bromo-4-hydroxybenzylidene)cyclopentanone* (**13**): Yellow microcrystals (8 mg, 2% yield) from hexanes/acetone. ^1^H-NMR (MeOD) δ 3.10 (s, 4H), 6.97 (d, *J* = 8.4 Hz, 2H), 7.39 (s, 2H), 7.48–7.50 (m, 2H), 7.79 (d, *J* = 1.3 Hz, 2H). LC *t* = 6.2 min. HRMS *m*/*z* Calcd. for C_19_H_15_O_3_Br_2_ 448.9388 [M + H]^+^, Found 448.9380.

*(2E,6E)-2,6-bis(3-bromo-4-hydroxybenzylidene)-4-methylcyclohexanone* (**14**): Yellow microneedles (43 mg, 9% yield) from dichloromethane/hexane. Solvent system B, elution at 100% dichloromethane. ^1^H-NMR (MeOD) δ 1.08–1.11 (m, 3H), 1.84–1.87 (m, 1H), 2.53 (*t*, *J* = 12.5 Hz, 2H), 2.99–3.01 (m, 2H), 6.94 (d, *J* = 8.4 Hz, 2H), 7.34 (d, *J* = 8.2 Hz, 2H), 7.59 (s, 2H), 7.63 (s, 2H). LC *t* = 6.2 min. HRMS *m*/*z* Calcd. for C_21_H_19_Br_2_O_3_ 476.9701 [M + H]^+^, Found 476.9701.

*Ethyl (3E,5E)-3,5-bis(3-bromo-4-hydroxybenzylidene)-4-oxocyclohexanecarboxylate* (**15**): Grey-green microneedles (747 mg, 14% yield) from dichloromethane/hexane. Solvent system B, elution at 5% MeOH. ^1^H-NMR (MeOD) δ 1.14 (*t*, *J* = 7.1 Hz, 2H), 2.83–2.86 (m, 1H), 3.08–3.31 (m, 4H), 4.08 (q, *J* = 7.1 Hz, 2H), 6.95 (d, *J* = 8.4 Hz, 2H), 7.34 (d, *J* = 8.1 Hz, 2H), 7.62 (s, 2H), 7.64 (s, 2H). LC *t* = 6.2 min. HRMS *m*/*z* Calcd. for C_23_H_21_Br_2_O_5_ 534.9756 [M + H]^+^, Found 534.9731.

*(2E,6E)-2,6-bis(3-Bromo-4-hydroxybenzylidene)-4-hydroxycyclohexanone* (**17**): Orange microneedles (131 mg, 3% yield) from dichloromethane/methanol. Solvent system B, elution at 4% MeOH. ^1^H-NMR (DMSO-*d*_6_) δ 2.84–2.88 (m, 2H), 3.00–3.03 (m, 2H), 3.99–4.00 (m, 1H), 7.03 (d, *J* = 8.2 Hz, 2H), 7.41 (d, *J* = 8.0 Hz, 2H), 7.53 (s, 2H), 7.69 (s, 2H), 10.79 (s, 2H). LC *t* = 5.6 min. HRMS *m*/*z* Calcd. for C_20_H_17_Br_2_O_4_ 478.9494 [M + H]^+^, Found 478.9511.

*(3E,5E)-3,5-bis(3-Bromo-4-hydroxybenzylidene)dihydro-2H-pyran-4(3H)-one* (**18**) [34]: Yellow microneedles (4 mg, 1% yield) from dichloromethane/hexane. Solvent system B, elution at 5% MeOH. ^1^H-NMR (MeOD) δ 3.35 (s, 4H), 6.96 (d, *J* = 8.4 Hz, 2H), 7.22 (dd, *J* = 8.4, 1.6 Hz, 2H), 7.52 (d, *J* = 1.5 Hz, 2H), 7.6 (s, 2H). LCMS *t* = 6.1 min, *m*/*z* Calcd. for C_19_H_15_Br_2_O_4_; C_19_H_14_Br_2_NaO_4_; C_38_H_28_Br_4_NaO_8_ 466.93, 464.93, 468.92; 488.91; 954.84 [M + H]^+^; [M + Na]^+^; [2M + Na]^2+^, Found 466.93, 464.93, 468.92; 488.91; 954.91.

*(3E,5E)-3,5-bis(3-Bromo-4-hydroxybenzylidene)-1-methylpiperidin-4-one* (**20**) [34]: Orange microcrystals (10 mg, 2% yield) from dichloromethane/hexane. Solvent system B, elution at 5% MeOH. ^1^H-NMR (MeOD) δ 2.51 (s, 3H), 3.81 (s, 4H), 6.96 (d, *J* = 8.4 Hz, 2H), 7.31 (d, *J* = 8.4 Hz, 2H), 7.60 (s, 2H), 7.64 (s, 2H). LCMS *t* = 5.4 min, *m*/*z* Calcd. for C_20_H_18_Br_2_NO_3_; C_20_H_17_Br_2_NNaO_3_; C_40_H_34_Br_4_N_2_NaO_6_ 479.96, 477.96, 481.96; 501.95; 980.00 [M + H]^+^; [M + Na]^+^; [2M + Na]^+^, Found 479.96, 477.95, 481.93; 501.92; 980.95.

#### 5.2.2. Claisen-Schmidt

Method B:

Piperidone hydrate hydrochloride (1.0 g, 6.5 mmol), was added to glacial acetic acid (40 mL) and saturated by a slow bubbling stream of HCl gas. 3-Bromo-4-hydroxybenzaldehyde (3.9 g, 19.5 mmol), was added to glacial acetic acid (50 mL) and then added to the piperidone solution. The reaction mixture was stirred at room temperature for 48 h, after which a yellow precipitate was filtered from the solution, washed with D.I. H_2_O (50 mL), EtOH (50 mL), diethyl ether (50 mL), and dried under vacuum [61].

*(3E,5E)-3,5-bis(3-Bromo-4-hydroxybenzylidene)-4-oxopiperidin-1-ium acetate* (**19**): Yellow microcrystals (2.4 g, 70% yield) from acetic acid. ^1^H-NMR (DMSO-*d*_6_) δ 3.40 (s, 4H), 4.47 (s, 3H), 7.13 (d, *J* = 8.4 Hz, 2H), 7.40 (d, *J* = 8.3 Hz, 2H), 7.3 (s, 2H), 7.75 (s, 2H), 9.57 (s, 1H), 11.20 (s, 1H). LC *t* = 5.4 min. HRMS *m*/*z* Calcd. for C_19_H_16_Br_2_NO_3_ 463.9497 [M + H]^+^, Found 463.9516.

Method C:

Ethyl *3E,5E*-bis(3-Bromo-4-hydroxybenzylidene)-4-oxocyclohexanecarboxylate (250 mg, 0.47 mmol) was hydrolyzed in a solution of THF (50 mL), H_2_O (25 mL), and NaOH (~5 eq), for 48 h. Aqueous HCl was added until pH was at congo red. THF was added until one layer was formed, and the precipitate product was collected by filtration.

*(3E,5E)-3,5-bis(3-Bromo-4-hydroxybenzylidene)-4-oxocyclohexanecarboxylic acid* (**16**): Yellow microcrystals (180 mg, 75% yield) from THF/H_2_O. ^1^H-NMR (MeOD) δ 2.78–2.81 (m, 1H), 3.05–3.10 (m, 2H), 3.20–3.31 (m, 2H), 6.96 (d, *J* = 8.4 Hz, 2H), 7.38 (d, *J* = 8.2 Hz, 2H), 7.64 (s, 2H), 7.67 (s, 2H). LC *t* = 5.8 min. HRMS *m*/*z* Calcd. for C_21_H_17_Br_2_O_5_ 506.9443, [M + H]^+^, Found 506.9465.

### 5.3. Cell Lines, Plasmids, and Antibodies

Human osteosarcoma U2OS and normal rat intestinal epithelial cells were maintained in Eagle’s minimal essential medium (DMEM) (Mediatech/Corning, Oneonta, NY, USA) and RPMI media, which were supplemented with 10% fetal bovine serum (Thermo Fisher Scientific, Waltham, MA, USA) and antibiotics. For p53 activation, Doxorubicin (Dox, Sigma, St. Louis, MO, USA) was used. The compounds were dissolved in DMSO (Sigma). The antibodies used for immunoprecipitation and immunoblotting were p53 (sc-6243), p21 (sc-397), and CBP (sc-7300) from Santa Cruz Biotechnology Inc. Dallas, TX, USA; p53Ser15p (9282), p53K382ac (2525), H2AX S139p (9718), H3S28p (9713), PARP (5625) and all Cyclins (4656 (A2), 12231 (B1), 4132(E2) antibodies from Cell Signaling Technology Inc., Danvers, MA, USA; H3 (ab1791), H3K27me3 (1791), H3K9ac (ab4441) and EZH2 (ab3748) antibodies from Abcam, Cambridge, MA, USA; and Actin (A4700) from Sigma.

### 5.4. Luciferase Assay for Structure Activity Relationship and IC_50_ Determination

A luciferase-based reporter assay in U2OS cells was developed to screen and to quantitate the inhibitory effects of newly synthesized *C*_5_-Curcumionoid analogues by using Dox-induced p53 response element system as well as to determine the respective IC_50_ values of the potential CBP HAT inhibitor. U2OS cells were co-transfected with p53 reporter element in tandem with luciferase gene (1 μg) and Renilla luciferase (100 ng) vectors in 24-well plate format using Fugene 6 (Promega, Madison, WI, USA). Briefly, total of 1.1 μg of vector was incubated with 3 mL of Fugene 6 reagent for 30 min. In these experiments, DMSO, transfected cells with empty vector and cells without Dox treatment were used as controls. DMSO concentration was maintained at 0.01%. Transfected cells with Dox treatment were used as positive control. The luciferase activity was estimated by following the manufacturer’s instruction (Promega, Madison, WI, USA) in a luminometer. Both active and passive lysis of cells yielded consistent results. The inhibitory activity (IC_50_) of a small molecule on luciferase activity was obtained from the average of three biological replicates using the PRISM software (La Jolla, CA, USA).

### 5.5. In Vitro HAT Assay

Following manufacturer’s instructions (Active Motif, Carlsbad, CA, USA), fluorescence-based HAT assay was performed using recombinant CBP and PCAF HAT enzymes [62,63]. Briefly, in presence of curcumin and NiCur, the reaction mixture was constituted with H3 peptide as a substrate and acetyl-CoA as a cofactor, which were incubated for 30 min. The reactions were quantitated by recording the excitation at 360–390 and emission at 450–470 nanometers.

### 5.6. Computational Analysis

Docking calculations used Autodock-4 [64] that searches simultaneously the space around the targeted site of the protein and the conformation of the ligand to find the complex with the lowest binding free energy, as expressed with a physico-chemically derived scoring function. The best docked pose was extracted with the program Dockres. The list of ligand–target contacts was obtained with the program Simulaid.

### 5.7. Immunoblotting

U2OS and normal rat intestinal epithelial cells were harvested cells and lysed in lysis buffer (20 mM Tris (pH 8.0), 150 mM NaCl, 1 mM EGTA, 1% Triton X-100, and 50 mM NaF) containing protease inhibitor cocktail (Sigma). The cells were sonicated and spun down at 14,000 rpm for 30 min at 4 °C. After protein estimation, 30–50 μg of lysates were subjected to SDS-PAGE, transferred onto nitrocellulose membranes, blocked with 5% milk/PBS and blotted with a primary antibody. Horseradish peroxidase (HRP)-labeled secondary antibodies (goat anti-mouse or anti-rabbit) were added for 60 min at room temperature, and the blots were washed with TBS (20 mM Tris, 150 mM NaCl, and 0.05% Tween-20) and subjected to autoradiography after development of reaction by ECL (GE Health Care, Pittsburg, OA, USA and LI-COR Biosciences, Lincoln, NE, USA) [16,17,18].

### 5.8. Chromatin Immunoprecipitation Quantitative PCR (ChIP-qPCR)

For ChIP-qPCR, EZ-Magna ChIP G (Millipore, Burlington MA, USA) was used and manufacturer’s instruction was followed. For ChIP analyses, U2OS cells were treated with Dox in presence or absence of NiCur. IP was carried out with antibodies to the following proteins: RNA polymerase II (2 μg), p53 (2 μg), CBP (2.5 μg), EZH2 (2.5 μg), H3 (6 μg), H3Kac (6 μg), H4Kac (6 μg), H3Kme3 and H3K27ac (2.5 μg). An aliquot was amplified and analyzed by qPCR using Human *CDKN1A* promoter primers (Cell Signaling Technology Inc., Danvers, MA, USA). The qPCR data was normalized using H3 antibody as a control with regard to the input.

### 5.9. BrdU Cell Proliferation Assay

To test the effects of NiCur on proliferation of U2OS and normal rat gastrointestinal epithelial cells, BrdU incorporation assay was performed in 96-well plates using calorimetry-based kit from Calbiochem Burlington Massachusetts, USA (Cat # QiA58). One hundred microliters of 5000–10,000 U2OS and rat gastrointestinal epithelial cells were seeded with DMEM and RPMI media (Corning, Oneonta, NY, USA) with 10% fetal bovine serum (Thermo Fisher Scientific, Waltham, MA, USA), respectively. After 16 h, cells were treated with 1.5 μM of NiCur and BrdU in presence or absence of 300 ng of Dox. To rule out the possibilities of any background effects, controls included in the assay were media alone, or together with DMSO and with BrdU and the untreated cells. Subsequently, after 24 h, cells were fixed, and then treated with anti-BrdU antibody. After washing, the wells were further incubated with secondary antibody labeled with HRP. After washings, the reaction in each well was developed using TMB as a substrate, and then finally, reaction in each well was stopped by adding stop solution and optical density was measured at 450 nm.

### 5.10. Cleaved Caspase 3 Apoptosis Assay

To test the effects of NiCur on apoptosis, intracellular levels of cleaved Caspase 3 was determined by using a kit from Thermo Fisher Scientific, Waltham, MA, USA (Cat# 62218) following manufacturer’s instructions with minor modifications. Approximately, 10,000 U2OS and normal rat gastrointestinal epithelial cells were seeded in 96 well plates. After overnight, cells were treated with 300 ng of Dox in presence and absence of 1.5 μM of NiCur. The controls included were media alone and untreated cells with and without NiCur. After 24 h of incubation, cells were fixed with 4.0% formaldehyde. After washing, cells were permeabilized and then each well was incubated in blocking solution to prevent non-specificity. After washing, the each well was incubated with anti-Caspase 3 antibody (Cell Signaling Technology Inc., Danvers, MA, USA; Cat# 9664), which can show reactivity to Caspase 3 proteins from multiple species, including humans and rats. Subsequently, each well was incubated with anti-rabbit HRP-labeled secondary antibodies. Finally, the reaction in each well was developed using TMB and the plate was read at 450 nm.

## Figures and Tables

**Table 1:** Screening for identifying potent CBP HAT inhibitors by determining the percent inhibition in Dox-mediated activation of p53 response element in U2OS cells treated with 500 nM of *cy*-*C*_5_-curcuminoid analogs with modifications on sides.

Compound ID	X	R^1^	R^2^	R^3^	R^4^	% Inhibition
DMSO	-	-	-	-	-	0
Curcumin	-	-	-	-	-	61
**1**	C	H	Br	OH	H	31
**2**	C	H	CN	H	H	>99
**3**	C	H	Br	OCH_3_	H	53
**4**	C	CH_3_	H	H	H	6
**5**	C	H	CH_3_	H	CH_3_	25
**6**	C	H	CH_3_	OH	CH_3_	40
**7**	C	H	H	OH	H	38
**8**	C	F	H	H	H	13
**9**	C	Br	H	H	H	17
**10**	C	H	Br	H	H	88
**11**	C	H	H	Br	H	4
**12**	N	H	- ^a^	H	H	>99

^a^ N lone pair.

**Table 2:** Screening for identifying potent CBP HAT inhibitors by determining the percent inhibition in Dox-mediated activation of p53 response element in U2OS cells treated with 500 nM of *cy*-*C*_5_-curcuminoid analogs with central ring modifications.

Compound ID	*n*	Y	Z	(%) Inhibition
DMSO	-	-	-	0
Curcumin	-	-	-	61
**1**	1	CH	H	31
**13**	0	-	-	39
**14**	1	CH	CH_3_	53
**15**	1	CH	CO_2_Et	82
**16**	1	CH	CO_2_H	26
**17**	1	CH	OH	26
**18**	1	O	- ^a^	22
**19**	1	N ^b^	H	>99
**20**	1	N	CH_3_	29

^a^ O Lone pair; ^b^ ammonium salt.

## Supplementary Materials

The following are available online, Table S1: Luciferase data and % inhibition of analogues with modifications on sides. Table S2: Luciferase data and % inhibition of analogues with modifications on central ring. Table S3: Normalization of p53, p53K382ac and p53S15p to Histone H3.
