# Supplementary material for: Inhibitor of CBP Histone Acetyltransferase Downregulates p53 Activation and Facilitates Methylation at Lysine 27 on Histone H3
Source: Molecules. 2018 Aug 2;23(8):1930. doi: 10.3390/molecules23081930 (PMC6222455; doi:10.3390/molecules23081930)
Supplement: Supplementary file 1 [file molecules-23-01930-s001.pdf]

Supplementary Information

Inhibitor of CBP Histone Acetyltransferase Downregulates p53 Activation and Facilitates Methylation at Lysine 27 on Histone H3

Supplementary Table S1: Luciferase data and % inhibition of analogues with side modifications.

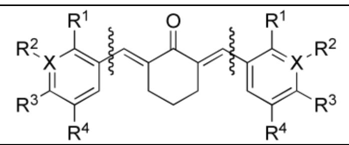

CPDID	Analogues	X	R¹	R²	R³	R⁴	Luc. Values*	%-Activation	%-Inhibition
**Cur#**	-	-	-	-	-	-	150	39	61
**1**	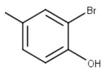	C	H	Br	OH	H	265	69	31
**2**	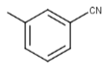	C	H	CN	H	H	-80	<1	>99
**3**	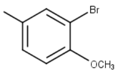	C	H	Br	OCH_3	H	180	47	53
**4**	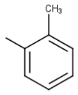	C	CH_3	H	H	H	360	94	6
**5**	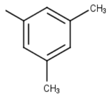	C	H	CH_3	H	CH_3	290	75	25
**6**	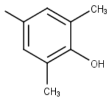	C	H	CH_3	OH	CH_3	230	60	40
**7**	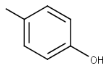	C	H	H	OH	H	240	62	38
**8**	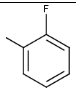	C	F	H	H	H	335	87	13
**9**	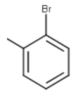	C	Br	H	H	H	320	83	17
**10**	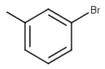	C	H	Br	H	H	45	12	88

**11**	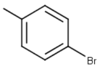	C	H	H	Br	H	370	96	4
**12**	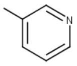	N	H	-[a]	H	H	-80	<1	>99

[*] Average Luciferase (Luc) values with background correction; [#] Curcumin; [a] N lone pair

Supplementary Table S2: Luciferase data and % inhibition of analogues with central ring modifications.

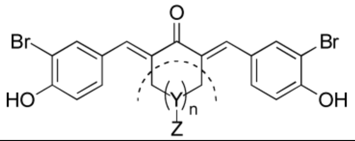

CPD ID	Analogues	n	Y	Z	Luc. Values*	%-Activation	%-Inhibition
**Cur**[#]	-	-	-	-	150	39	61
**1**	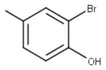	1	CH	H	265	69	31
**13**	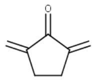	0	-	-	235	61	39
**14**	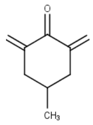	1	CH	CH_3	180	47	53
**15**	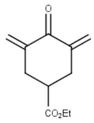	1	CH	CO_2Et	70	18	82
**16**	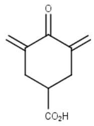	1	CH	CO_2H	285	74	26
**17**	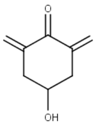	1	CH	OH	285	74	26
**18**	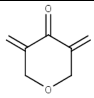	1	O	-[a]	300	78	22
**19**	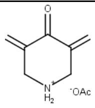	1	N[b]	H	-85	<1	>99
**20**	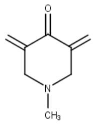	1	N	CH_3	275	71	29

*Average Luciferase values with background correction; [#] Curcumin; [a]O lone pair; [b]ammonium salt

The Percent inhibition from three biological with technical repeats was calculated as described below:

Luciferase value without Dox treatment (Background) = 90; **Luciferase Value with Dox treatment alone (Positive control)** = 475; Background correction[#]= 475 - 90= 385; Background correction was done for each compound; **% Activation =** 100*Luciferase values of analogues/385; **% Inhibition** =

100 – % Activation; **Or % Inhibition of Analogues**= 100* Luciferase Value of Positive control - Luciferase Value of Analogues/Positive Control

Supplementary Table S3

Normalization of p53, p53K382ac and p53S15p to Histone H3

**Normalized to Histone H3**					
**Antibodies**	**UT**	**DOX**	**0.5 μM**	**1.0 μM**	**1.5 μM**
**Histone H3**		**1**	**1**	**1**	**1**
**IP: p53 IB:p53**		0.5	0.4	0.35	0.3
**IP: p53 IB:p53S15p**		0.5	0.45	0.4	0.3
**IP: p53 IB:p53K382ac**		0.4	0.3	0.1	0.04

In the above table, the columns represent the experimental conditions with respect to the treatments with Dox and NiCur. The rows represent the values of IB signals obtained by indicated antibodies on the left column respectively. The density of immunoblot bands with different antibodies were calculated with Adobe Photoshop. The density obtained for histone H3 for each condition was normalized to 1. Subsequently, the density values calculated for p53, p53S15p and p53K382ac were normalized with Histone H3 respectively.
